# Metal Organic Frameworks as Drug Targeting Delivery Vehicles in the Treatment of Cancer

**DOI:** 10.3390/pharmaceutics12030232

**Published:** 2020-03-05

**Authors:** Mengru Cai, Gongsen Chen, Liuying Qin, Changhai Qu, Xiaoxv Dong, Jian Ni, Xingbin Yin

**Affiliations:** 1 School of Chinese Material Medical, Beijing University of Chinese Medicine, Beijing 102488, China; cmrtcm@bucm.edu.cn (M.C.); 20170931912@bucm.edu.cn (G.C.); 20180935094@bucm.edu.cn (L.Q.); quchanghai@bucm.edu.cn (C.Q.); 201801020@bucm.edu.cn (X.D.); 2 Beijing Research Institute of Chinese Medicine, Beijing University of Chinese Medicine, Beijing 100029, China

**Keywords:** metal organic framework, targeting drug delivery system, nanoparticle

## Abstract

In recent years, metal organic frameworks (MOFs) have been widely developed as vehicles for the effective delivery of drugs to tumor tissues. Due to the high loading capacity and excellent biocompatibility of MOFs, they provide an unprecedented opportunity for the treatment of cancer. However, drugs which are commonly used to treat cancer often cause side effects in normal tissue accumulation. Therefore, the strategy of drug targeting delivery based on MOFs has excellent research significance. Here, we introduce several intelligent targeted drug delivery systems based on MOFs and their characteristics as drug-loading systems, and the challenges of MOFs are discussed. This article covers the following types of MOFs: Isoreticular Metal Organic Frameworks (IRMOFs), Materials of Institute Lavoisier (MILs), Zeolitic Imidazolate Frameworks (ZIFs), University of Oslo (UiOs), and MOFs-based core-shell structures. Generally, MOFs can be reasonably controlled at the nanometer size to effectively achieve passive targeting. In addition, different ligands can be modified on MOFs for active or physicochemical targeting. On the one hand, the targeting strategy can improve the concentration of the drugs at the tumor site to improve the efficacy, on the other hand, it can avoid the release of the drugs in normal tissues to improve safety. Despite the challenges of clinical application of MOFs, MOFs have a number of advantages as a kind of smart delivery vehicle, which offer possibilities for clinical applications.

## 1. Introduction

Cancer is one of the major diseases to human health and the morbidity increased gradually. Although treatments of cancer were being improved and survival rates increased in recent years, the heterogeneity of cancer still demands further therapeutic strategies [1]. The most common cancer treatments are restricted to chemotherapy, radiation, and surgery, involving a lot of side effects caused by a non-specific tissue distribution of anticancer agents, insufficient drug concentrations at the cancer and unmanageable toxicity. In recent years, a new class of cancer treatment methods, immunotherapy, has a higher anti-cancer effect, but it is more toxic and only effective for some patients [2]. Cancer targeting is one of the newly appeared promising biotherapies of cancer. The system of targeting anticancer drug to the tumor tissues could improve local drug concentration, enhance the curative effects, and reduce the side effects remarkably. To selectively target drugs to tumor tissues, a technique called targeting drug delivery system (TDDS) is used [3]. TDDS plays a major role in the treatment of cancer. It uses a variety of vehicles, such as liposomes, microspheres, nanoparticles, microemulsion, albumin, lipoproteins, emulsion, and polymer conjugates. Its development, to a great extent, depends on the design of nanocarrier materials. Chitosan, hyaluronic acid (HA), polyethylene glycol (PEG), lipoprotein, human serum albumin (HSA), graphene, mesoporous silica nanoparticles (MSNs) and metal organic frameworks (MOFs) are commonly used [4,5]. With the development of polymers and materials, drug nanocarrier materials have received much attention. 

Metal organic frameworks (MOFs) are a class of hybrid materials formed by the self-assembly of metal ions or clusters and polydentate bridging ligands typically under mild conditions [6]. Compared with the traditional porous material, MOFs have many advantages: numerous categories (at present, there are more than five thousand kinds of materials, and the quantity that can be synthesized in theory is almost infinite) [7], multiple functions (due to virtually limitless combinations of metals and ligands, the physicochemical properties of MOFs can be judiciously tuned for specific applications), the porosity and specific surface area are large, the crystal density is small, controllable pore size, good biocompatibility, and bionic catalytic properties [8]. MOFs have shown promise for a number of diverse applications including gas storage, catalysis, nonlinear optics, separations, sensing, and light-harvesting. 

MOFs can be regarded as potential drug delivery nanovehicles because of the abilities of their adjustable pore size, high surface area, and the possibility to add functional groups to the frameworks. MOFs can carry huge amounts of drugs, therefore biomedical applications of MOFs have focused on their use as anticancer drug delivery vehicles. Since this research field is rapidly expanding, more publications are reported about complex nanotheranostics. We were motivated to give an over-view of such modern attractive nanosystems, along with an outline of the research field in general. In this article, the recent developments on MOFs as targeting drug delivery systems which are able to release therapeutic compounds once they reached the diseased tissues and cells are reviewed. 

## 2. Synthesis, Functionalization, and Biomedical Applications of MOFs 

### 2.1. MOFs Synthesis and Functionalization

So far, many synthetic methods of MOFs have been reported, such as the solvothermal method, rapid precipitation method, one-pot synthesis, reverse microemulsion, a rapid microwave-assisted method, ultrasonic synthesis, and so on. The synthesis methods and drug loading characteristics of different MOFs are listed in Table 1. Several types of MOFs are discussed in this article. Table 2 categorizes MOFs and lists representative MOFs.

Usually, metal organic frameworks are synthesized by solvothermal method, which is one of the most classical methods for the synthesis of MOFs. For instance, Yang and coworkers used solvothermal method to synthesize IRMOF-3 [9]. The folic acid (FA) was then modified on IRMOF-3 by post-synthesis modification. Similarly, Angshuman et al. utilized a mixed solvent solvothermal method to get Fe_3_O_4_@IRMOF-3 [10]. The material was placed in a mixed solvent of DMF and pure ethanol containing PVP, and then heated at 100 °C for 4 h to obtain a dark brown nano materials. Particle size of synthetic IRMOF-3 was less than 100nm, and the particle size of Fe_3_O_4_@IRMOF-3 was about 200 nm. The hydrophobic nano-platform encapsulated paclitaxel, which had a drug loading of 12.32% and released 65% under physiological conditions for 4 days. In addition, the brown N_3_-UiO-66-NH_2_ was synthesized by Nian using solvothermal method [11]. These nanocrystals were consistent in size and had good drug loading properties. However, sometimes nanoparticles synthesized by solvothermal method may have a large particle size, which is not conducive to targeted administration by post-synthesis modification. Therefore, we need to properly control the ratio of metal ions to organic ligands and the conditions under which the reaction is made to control the size of the particles, thereby promoting the functionalization of the drug-loaded particles. In the end, we still need to use some characterization methods (particle size distribution, scanning electron microscope, transmission electron microscope) to evaluate the dispersity of the nanoparticles.

**Figure 1:**
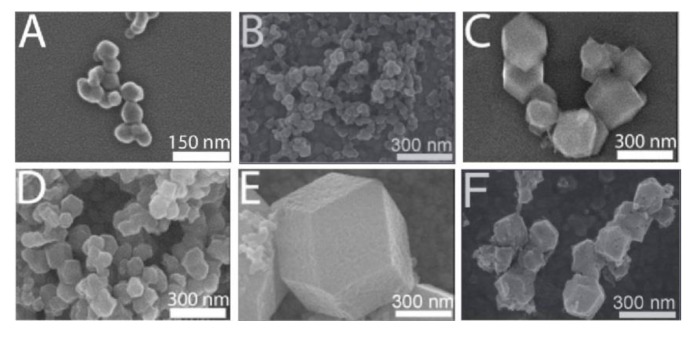
ZIF-90 nanoparticles (NPs) synthesized in trioctylamine at (**A**) 0 °C, (**C**) 100 °C, and (**E**) 150 °C and particles synthesized at room temperature using (**B**) trioctylamine, (**D**) tributylamine, (**F**) trimethylamine [12]. Reprinted (adapted) with permission from (Versatile Synthesis and Fluorescent Labeling of ZIF-90 Nanoparticles for Biomedical Applications). Copyright (2016) American Chemical Society.

The nanoparticles with smaller particle size can be synthesized by the rapid precipitation method, but the nanoparticles obtained by the method are usually cluster-like, have no fixed crystal form, and rarely obtain single crystal. Christopher synthesized ZIF-90 with different particle sizes (60–90 nm, 200–300 nm, 100–200 nm) by adding different amines (trioctylamine, tributylamine, trimethylamine) (Figure 1) [12]. This method can quickly synthesize metal organic framework particles. As the reaction temperature increases, the particle size also increases gradually. This method can rapidly synthesize MOF particles, usually constituting a precipitate at the moment of amine addition. 

One-pot synthesis method is also widely developed in the preparation of metal organic frameworks. The most well-known ZIF-8 can be synthesized in this way. Shi prepared CQ@ZIF-8 by one-pot method with a drug-loading of 18% [13]. The particles were regular octahedral structures with an average particle size of 250 nm. ZIF-8 is consistent under physiological conditions and is easily degraded under acidic conditions. These features are beneficial to the targeted transport of the nanoparticles. Coincidentally, Song also synthesized the photosensitizing target formulation ZnPc@ZIF-8/CTAB by the same method, with a drug-loading of 29.5% [14]. After the release of the photosensitizer ZnPc, the intracellular reactive oxygen species increased, thereby producing an anticancer effect. The one-step synthesis of ZIF-8-based nano drug loading systems is appropriate and can target weak acidic environments, and it has attracted much attention as a host for delivering both hydrophilic and hydrophobic drugs. In addition, one-pot synthesis was administered by Wang et al. who obtained the tumor targeting MOF of pH response and redox response (DOX@TTMOF) [15]. Additionally, Shi and coworkers developed Ce-MOF by one-step synthesis [16]. They then combined ATP aptamer to the Ce-MOF modified on the bare gold electrode. ATP aptamer could detect serum ATP in tumor patients by electrochemical impedance spectroscopy. The precise diagnosis of tumors is the basis of accurate tumor treatment. In addition, Su et al. obtained UiO-66@AgNCs@Apt@DOX by one-pot encapsulation, who’s loading efficiency was twice that of two separate processes [17]. All of the above demonstrate the simplicity and efficiency of one-step synthesis. One-step synthesis has been the choice of most researchers for the development of drug-loaded MOFs.

However, when a part of the metal organic frameworks was used for drug loading, it is often requested that the drugs have a strong interaction with the carrier to achieve a high drug loading capacity. For example, ZIF-8 can be successfully used in one-step synthesis and drug delivery only if the transported drugs have an acidic group. However, not all effective drugs have acid groups, which greatly affects the use of ZIF-8 as a drug carrier. Therefore, a strategy is urgently needed to compensate for this shortcoming. Zhang et al. wanted to load Cytarabine (Ara) with ZIF-8, but because of the lack of a drug-acting group, it was not possible to achieve high drug loading capacity [18]. Therefore, they proposed that Cytarabine (Ara) combined with New indocyanine green (IR820) to form a prodrug together encapsulated in ZIF-8, this strategy greatly increased the drug loading capacity, which suggests that we can strengthen affinity between the carriers and the drugs by structural modification or other methods to increase the drug loading capacity of MOFs.

Other methods are also provided for the synthesis of metal organic frameworks. Zhang and his group used vigorously stirring in combination with ultrasonic condition to synthesize ZIF-90 [19]. In this way, they synthesized ZIF-90 with a particle size of less than 300 nm to serve as drug carriers. They used ZIF-90 to load both 5-FU and DOX, which improved the efficacy and overcame the problem of drug resistance. In addition, this drug delivery system could rely on pH for targeted drug delivery. A rapid microwave-assisted method was utilized by Jia et al. who synthesized MB@THA-NMOF-76@cRGD (HTHA = 4,4,4-trifluoro-1-(9-hexylcarbazol-3-yl)-1,3-butanedione, MB = methylene blue, NMOF = nanoscale metal−organic framework, and cRGD = cyclic ArgGly-Asp peptide) [20]. This drug loading system has an average particle size of 89 nm and good uniformity. Through x-ray diffraction, scanning electron microscope, and thermogravimetric analysis, it was proved that the components of the system were modified. The author also studied its stability and found that the system is highly resistant to light and acid. The liquid-solid-solution (LSS) method was reported by Cai who synthesized Fe-soc-MOF to achieve photothermal therapy [21]. The nanosystem has a particle size of about 100 nanometers, which is much smaller than those synthesized by other methods. Another unique approach was introduced by Yu et al. who employed a template-directed synthesis strategy [22]. In brief, this strategy relied on the growth of skeletal and interconnected ZIF-8 crystals on a long and soft filamentous micelle, which was finally removed by extraction to obtain zif-8 hollow nanotubes. This nanotube had an ultra-high drug loading rate of 350% and was effective in avoiding the reticuloendothelial system (RES), forming a long-acting cycle (about one week). Cao used a surfactant to assist synthesize ZIF-8 hollow nanospheres and encapsulated 10-HydroxyCamptothecin (HCPT) for tumor therapy [23]. It provided a new idea for the synthesis of MOFs.

With the in-depth study of MOFs, single nanocarriers often have certain defects, accompanied by low drug loading, burst release, and so on. The poor biocompatibility of some metal organic frameworks also limits their clinical application. In recent years, researchers have worked to solve these problems. In this context, composite nanocarriers have been shown to be better for the treatment of cancer. For example, MnCo-MOF has strong toxicity and its application in vivo is dangerous. Wang and colleagues developed a polydopamine hybrid nanogels [24]. The nanosystem could effectively reduce the toxicity of the MOFs and improve the photothermal conversion efficiency of the photosensitizer. In vitro and in vivo experiments show that the materials had good biocompatibility and excellent photothermal effect. This strategy suggests that we can extend it to therapeutic applications of other MOFs. Other than this, Abhik and coworkers studied the effects of complexes of Fe_3_O_4_ nanoparticles and MOFs on the drug loading and releasing behaviors [25]. They found that the drug loading of the composite was higher than that of a single nanocarrier. The materials had not burst release behavior, and the loaded doxorubicin could be released for 25 days. Even in the first few days, Fe_3_O_4_@MIL-100 did not have any sudden release behavior. All the above experiments are excellent examples of MOFs-based composite materials for targeted anti-cancer treatment. This indicates that we can further develop multifunctional composite materials based on MOFs to conduct anti-cancer research at a higher level.

### 2.2. Properties of Metal Organic Frameworks Regarding Drug Delivery Applications

The metal organic frameworks usually have a large specific surface area, a large pore diameter, good biocompatibility, non-toxic to the human body and easy to be metabolized. Therefore, MOFs are suitable as carriers for drug delivery. In order to increase the drug loading capacity, control the rate of drug release, and deliver the drug to the destination accurately, we need to rationally adjust the pore size, particle size, stability, and other properties of the MOFs. Here, we only briefly introduce the three basic characteristics of MOFs that need to consider as drug carriers.

#### 2.2.1. The Effect of Pore Size of MOFs on the Drug Loading Capacity

Although the pore size of MOFs is adjustable, its regulation ability is restricted. Generally, a larger pore size means a higher drug loading capacity. In recent years, some researchers have prepared hollow MOFs to pursuit higher drug loading capacity and multi-functional targeted drug delivery. For example, Gao et al. synthesized hollow ZIF-8, which had a drug loading capacity of 51% and modified three substances [32]. In addition, we can also modify the pore size of MOFs by changing organic ligands to improve drug loading capacity. In recent years, there have been few studies in this field, which provides a new direction for our future research, to study the effects of metal organic framework nanoparticles with different pore sizes on drug loading performance.

#### 2.2.2. Particle Size Control to Achieve Functional Transfer of MOFs and Improve Biocompatibility

Particle size is another important property of drug-loaded MOFs. The particle size can determine the targeting ability of the drug delivery system. When the particle size is around 100 nm, the drug loading system is relatively easy to passively target to cancerous tissues. This phenomenon will be described in detail later. In order to achieve active or multi-functional targeting, the particle size of metal organic frameworks should preferably be within 100 nm to avoid clearance by macrophages of the reticuloendothelial system and liver. Therefore, it is necessary to control the particle size of MOFs. In a typical example, Duan et al. devoted research to controlling the particle size of AZIF-8 (amorphous zeolitic imidazolate framework-8) by a simple method and studying the effects of its particle size on the treatment of tumors [37]. They used nontoxic poly-allylamine hydrochloride (PAH) to precisely control the size of the AZIF-8 by one-pot synthesis method, which broke the tradition of being unable to control the MOFs’ particle size precisely and the need for toxic solvents for synthesis and modification. The addition of PAH could change the nucleation rate of AZIF-8, which affected the particle size of AZIF-8. The more PAH, the larger the particle size of AZIF-8, but the other physical and chemical properties were not covered. Through a series of in vitro and in vivo studies, AZIF-8 at 60 nanometers had the best curative effects with excellent biocompatibility and high tumor uptake capacity.

Gao needed to perform magnetic sensitization, light sensitivity, active targeting, and load chemotherapeutic drugs at the same time [31]. Thus, they studied the factors influencing the particle size. They concluded that the lower the concentration of the reactants, the larger the particle size of Fe-MIL-53-NH_2_. Additionally, Gao et al. studied the effects of benzoic acid on the particle size of UIO-66-NH_2_ and synthesized it by a hydrothermal method [38]. Unlike the above, the lower the benzoic acid, the smaller the particle size. Therefore, these experiments also reflected the effect of reactant concentration on the particle size of MOFs. However, these studies only proved the factors affecting particle size, and there lacked the study of particle size for effectiveness and safety. Some studies only considered the drug-loading capacity, but ignored the effects of particle size on the circulation in the body. Even with the highest drug-loading, the drug loading system is rapidly metabolized and even causes death of model animals after entering the systemic circulation. Therefore, the therapeutic effects cannot be achieved. Such researches are obviously lacking in value. In recent years, MOFs-based nanocarriers used for cancer treatment have a large difference in particle size, and there is a lack of comprehensive evaluation of the particle size in their application. This also provides direction for our future researches.

#### 2.2.3. Stability of MOFs: Another Property to Consider

Stability is the most basic requirement for the drug-loading systems. In order to improve the efficacy and reduce the toxicity of anticancer drugs, some intelligent drug-loaded nanosystems have begun to attract people’s attention. First, these nanoparticles should be stable when stored in vitro to ensure efficacy and safety. Then these drug-loaded nanosystems in vivo are preferably stable prior to reaching the target site and responsive to release of the drug at the tumor site. Rachel obtained Zn-MTX NCP (MTX = methotrexate, NCP = nanoscale coordination polymers) and Zr-MTX NCP materials using Zn^2+^ and Zr^4+^ as metal ions, respectively, using the high-temperature surfactant-assisted method and the microwave heating method, and found that both were unstable [30]. The reason might be that the particles would polymerize in the water to break the surface liposome. Finally, they employed microwave heating method to obtain Gd-MTX NCP. Then the phospholipid bilayer was utilized to wrap the metal organic framework. It was noted that the drug-loading system was stable. Therefore, the establishment of any drug-loading system should take into full consideration the influence of each element on its stability. However, more research is needed on the effects of stability on the body’s efficacy and toxicology.

## 3. Applications of Metal Organic Frameworks in Targeting Cancer

Normal cells rely on the integrity of regulatory circuits that control cell proliferation and maintenance. The regulatory circuits are disrupted in cancer cells, and the type and behavior of the cancer cell vary depending on the type of damage caused to the regulatory circuits [39]. This particularity can be exploited against tumor cells in attempts to treat the disease, using passive targeting, active targeting, physicochemical targeting, or a combination of the three [40].

**Figure 2:**
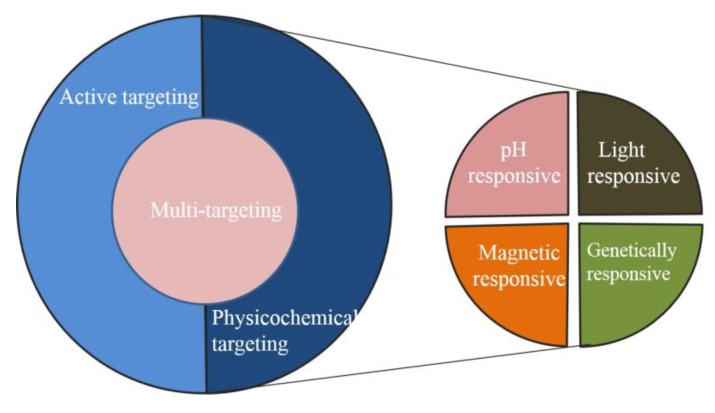
Schematic diagram of the types of tumor-targeted treatment strategies based on MOFs.

The use of targeted MOFs can also solve the lack of selectivity of some drugs, since they can host, transport and direct the therapeutic agents to the tumor selectively. This strategy permits to allow for a reduction in the dose required for conventional chemotherapy, increasing therapeutic efficacy and diminishing undesired side effects. Specific cells and organs within the body can also be targeted by modifying the nanomaterials’ surface with antibodies or appropriate ligands. For example, Chen et al. reported the Zr-UiO-66 was further functionalized with pyrene-derived polyethylene glycol (Py−PGA-PEG) and conjugated with a peptide ligand (F_3_) to nucleolin for targeting of triple-negative breast tumors [41]. Functionalized Zr-UiO-66 demonstrated strong radiochemical and material stability in different biological media. Based on the findings from cellular targeting and in vivo positron emission tomography (PET) imaging, the author concludes that Zr-UiO-66/Py−PGA-PEG-F_3_ can serve as an image-guidable, tumor-selective cargo delivery nanoplatform. Figure 2 summarizes the types of tumor targeted therapies. Table 3 summarizes the strategies for targeted therapy using MOFs in recent years.

### 3.1. Passive Targeted Therapy Used Metal Organic Frameworks

Passive targeting refers to the targeting of nano-targeted drug system on specific organs or disease sites according to the physiological mechanism after entering the blood circulation through intravenous injection. The nanoparticles were widely used in the anti-tumor drugs delivery system, because they have an ability to target tumor tissue passively, which is due to the enhanced permeation and retention effect (EPR effect.). Jihye et al. studied the passive targeting function of PCN-224 with different particle sizes [56]. They verified that MOFs can enhance photodynamic efficacy. Photosensitizers without MOFs get the lowest cytotoxicity. TCPP@PCN-24 with a particle size of 90 nm has the best photodynamic therapy effect, and TCPP@PCN-24 with a particle size of 190nm has the worst effect. Duan also proved that 60nm AZIF-8 has better anti-tumor effect than other particle sizes, due to the strongest retention effect in the tumor area of this particle size [37].

### 3.2. Active Targeted Therapy Based on Metal Organic Frameworks

Active targeted therapy is to transport the drug system to a specific part by means of the high affinity between the ligands and the overexpressed receptors on the targeted cell surface. Researchers can generally modify the surface of drug-loaded MOFs so that it is not recognized by macrophages. In addition, researchers can attach specific ligands (such as folate, RGD peptide, aptamers, etc.) on metal organic frameworks to target receptors (folate receptors, etc.). In addition, MOFs linked by monoclonal antibodies become immune microspheres to avoid macrophage uptake. Furthermore, researchers can modify MOFs into pharmacologically inert physics and activate them when they reach the surrounding cancer cells, so as to exert pharmacodynamic effect.

Modification of MOFs with folic acid is currently the most commonly used for targeted therapy. Since folate receptors are overexpressed on the surface of cancer cells. Folic acid can specifically bind to them, and then the drugs are focused and released in cancerous tissues. For example, Jihye and coworkers used folic acid to modify TCPP@PCN-224 which improved the efficacy of the original nano drug delivery system [56]. Same as above, folic acid was modified by Li et al. on a metal organic framework to get FA/DOX@UiO-68 [57]. They injected different substances into the tail vein of liver cancer mice. Tumors in the FA/DOX@UiO-68 group were smaller than those in the doxorubicin. Targeted anticancer activity of the nanosystem was confirmed by internal and external stimuli responses. Laha and his companions obtained IRMOF-3@CCM@FA in a one-step process, which also utilized folic acid to deliver curcumin to the triple negative breast cancer cells [58]. A series of in vivo and in vitro experiments demonstrated the superior targeting performance of this strategy.

Of course, there are some other overexpressed receptors on the surface of cancer cells, and researchers can use any of these receptors for active targeted therapy. For example, anisamide (AA) can specifically recognize sigma receptors on the surface of cancer cells. Based on this principle, Rachel used A DOPE-AA (DOPE = dioleoyl l-α-phosphatidyl-ethanolamine) in combination with Gd-MTX NCPs to kill leukemia cancer cells [30]. In addition, Hyaluronic acid specifically recognized overexpressed CD44 on the surface of tumor cells [50]. Therefore, MIL-100 (Fe) nanoparticles could aggregate in tumor tissues by modifying hyaluronic acid on their surfaces. MOF@HA@ICG NPs showed better photothermal effect at the tumor site by comparison with ICG and non-hyaluronic acid-modified drug-loaded MOFs (MOF@ICG NPs). Similarly, taken into account this strategy, Chen and colleagues have also developed VEGF (vascular endothelial growth factor)-responsive doxorubicin-loaded NMOFs, and the experimental results were also satisfactory [59]. This gating strategy has inspired researchers to find other suitable ligands for targeted therapies. More importantly, this strategy is not confined to the treatment of tumors, and other difficult diseases that have overexpressed receptors are applicable. Hu and coworkers synthesized Rho-BSA/Cu/NQ nanoparticles (NPs) based on albumin as a reactor for a simple method [35]. In particular, the nanoplatform had good solubility, therefore it may be used for injection administration. Owing to its ability to actively target, the system can have a high utilization rate at the target site. Based on a large number of previous studies, Hu has conducted a series of in vitro and in vivo experiments to demonstrate the potential for clinical application of the drug delivery system with highly effective utilization, excellent stability, superior biosafety. The success of these researches suggests that researchers can load any other applicable drug into this system for efficient cancer treatment.

Qi et al. modified anti-EpCAM antibodies to ZnMOFs, which specifically capture tumor cells [60]. Additionally, in Li’s report, a biomimetic theranostic O_2_-meter was introduced to people [61]. It referred to the modification of tumor cell membrane fragments on the surface of MOFs, so that it could identify tumor tissues efficiently, and could avoid the phagocytosis of macrophages and achieve immune escape. The biomimetic nanosystem will be described in detail later.

There are numerous specific receptors on the surface of cancer cells that can be used as targets for tumor diagnosis and treatment. The active targeting strategy is simple and easy, coupled with the unique affinity for tumor cells, greatly increasing the efficiency of targeted therapy. Therefore, active targeted therapy has become an indispensable part of anti-cancer research based on MOFs in recent years.

### 3.3. Physicochemical Targeting Depended on Metal Organic Frameworks

Physicochemical targeting refers to the use of physical or chemical methods to make the MOFs-based nano-drug delivery system release drugs or generate heat, thus leading to the apoptosis of tumor cells or the ablation of solid tumors. Without specificity, this method has few side effects on systemic tissues, and mainly plays a role in the lesions. Numerous scientists have carried out in-depth research on it.

#### 3.3.1. Metal Organic Frameworks for pH-Responsive Targeted Treatment

The rapid proliferation of tumor cells leads to local hypoxia, and its metabolism is also affected. The most direct result is an increase in lactate secretion and a decrease in pH. As the cancer tissue has a lower pH than normal tissue of the human body, the pH-responsive metal organic frameworks have become one of the most widely used environmentally responsive carriers.

ZIF-8 is one of the most classic metal organic frameworks because it releases the drugs in a tumor environment (lower pH) and remains stable under normal physiological conditions, with the characteristics of innocuity and good biocompatibility. In addition, ZIF-8 is usually synthesized and loaded drug by one-pot method, which is convenient and efficient. Thus, it attracts more interest of many researchers. For example, Chen loaded 3-methyladenine (3-MA) into ZIF-8 by a one-step method [62]. 3-MA is an autophagy inhibitor that interferes with autophagy behavior of tumor cells and induces tumor cells apoptosis. Owing to the sensitivity of ZIF-8 to pH, 3-MA is mainly released near tumor tissues, avoiding early metabolism and improving the bioavailability of 3-MA. In addition, Zheng et al. encapsulated the anti-cancer drug doxorubicin (DOX) within the ZIF-8 crystals [26]. ZIF-8 crystals loaded with DOX are efficient drug delivery systems in breast cancer therapy using pH-responsive release. The drug is released slowly at low pH (5.0–6.5) and not responded under physiological conditions (PBS, pH 7.4). However, drug resistance is one of the causes of chemotherapy failure in cancer. Therefore, several drugs have been used in combination for chemotherapy, which can improve the efficacy and reduce drug resistance. In addition, Zhang and his colleagues first reported a combination of doxorubicin and verapamil hydrochloride based on metal organic frameworks to defeat drug resistance [27]. Simultaneously, they modified the nano-drug delivery system with PEG-FA, which not only realized active targeting but also satisfied the long-term circulation. This PEG-FA/(DOX+VER)@ZIF-8 released faster and more at pH 5.0 than at physiological conditions. Furthermore, Liang et al. encapsulated doxorubicin and bovine serum albumin with ZIF-8, which also could be kept under physiological conditions and released under tumor conditions [63]. Tang et al. synthesizes a nanocapsule using the pH sensitivity of ZIF-8 [64].

In addition, countless other metal organic frameworks have also been found to have pH responsiveness. Wang and coworkers developed a pH-responsive core-shell metal organic framework (CS-MOFs) that degraded and released artesunate (AS) and Fe^3+^ at low pH for co-treatment of cancer [65]. After the release of Fe^3+^ is reduced to Fe^2+^, reacting with AS to form excess reactive oxygen species which leaded to enhanced cytotoxicity. Vandana synthesized MIL-101-Fe with pH responsiveness by solvothermal method [66]. This property facilitated the concentrated release of nanoparticles in the tumor area. Then they modified polyethyleneglycol (PEG) on the surface of the particles and found that it could enhance the stability of the nanoparticles. Chen et al. received 5-Fu @Dy(III)- organic framework which can release 78% at pH 5 but 50% at pH 7.4 [67].

Furthermore, a pH responsive nanoplatform can also be obtained by modification of the surface of the MOFs. It is a great choice to fix chitosan on the surface of MOFs. The amino group of chitosan can be protonated or deprotonated to achieve pH response [68]. Take into account this property, Reza and colleagues synthesized CS/DOX@Bio-MOF [69], which proved that the nanoplatform was almost completely released at pH 6.8 and released a little at pH 7.4. They have demonstrated that the nanoparticles have good biocompatibility. This report gives us a new idea for the preparation of pH-responsive nanoplatforms.

The pH-responsive nano drug-loading system based on MOFs can transport the drug to the tumor region with lower pH, improve the stability of the drug under physiological conditions, and enhance the release of the drug in the target region. However, this strategy does not completely prevent the release of the drug during its circulation in the body. Usually the drugs are released faster under low pH conditions, but they are also released under normal conditions, so it is necessary in order to further improve the system’s ability to selectively release the drug, or to improve the targeting ability by combining with other strategies.

#### 3.3.2. Light-Responsive Targeting of Cancer with MOF-Based Nano-Therapeutics

Recently, new treatment method based on photosensitized therapy for cancer has begun to receive attention of researchers and it is considered to be a promising treatment due to their many irreplaceable advantages, such as acting on local tumor sites, strong damage to the tumor, and no drug resistance. Phototherapy includes photothermal therapy (PTT) and photodynamic therapy (PDT) as well fluorescence imaging strategy.

Here, we first introduce several commonly used drugs and materials for photoresponsive therapy. As a kind of chromophores, porphyrin absorbs visible light, producing a singlet excited state that decays to the first triplet excited state [70]. This last state transfers its energy to molecular oxygen (^3^O_2_) in the medium, generating singlet excited oxygen (^1^O_2_), which is responsible for the death of cancer cells. Upconversion nanoparticles (UCNPs) are a class of materials that satisfy the anti-Stokes law of illumination. In other words, the materials can be pleased to be emit high-energy light by low-energy light, such as infrared light to excite visible light. They can be used for biomonitoring, medical treatment, CT and MRI. Currently, researchers in cancer treatment have a tendency to link this material with other carrier materials to meet high-efficiency diagnosis and treatment. This review will introduce UCNPs in combination with MOFs for targeting cancer in Section 3.4 more elaborate.

Fluorescence imaging is a sensitive photochemical reaction. Based on MOFs, this method can easily and accurately diagnose cancer while reducing the side effects on the body. This is expected to achieve the diagnosis and treatment of tumors in one step. Fluorescence imaging has witnessed significant advances in in vitro and in vivo imaging. Zhang and his peers used fluorescence reactions to diagnose cancer [53]. Specifically, Glucose oxidase was encapsulated in ZIF-8 (GOx/ZIF-8 composite), and then the drug system is modified by streptavidin to identify galectin-4, a tumor marker. Glucose oxidase produces hydrogen peroxide and further reacts with iron (II) ions to produce hydroxyl groups, which are recognized by gold nanoclusters to produce fluorescence quenching. Fluorescence intensity correlates with the concentration of galectin-4, even a very small amount of galectin-4 can be diagnosed. It provides a very sensitive way to detect tumors.

Photothermal therapy (PTT) refers to a method of targeting a material with high photothermal conversion efficiency to tumor tissue, and converting light energy into heat energy to kill cancer cells under the illumination of near-infrared light. Zhang et al. designed a multifunctional photosensitized nanoprobe [43]. Their group firstly formed a TMPyP@MOF by one-pot synthesis method, then Cy3-labeled caspase-3 substrate peptide and a FA were assembled on TMPyP@MOF surface. Thus, this nanoprobe could be targeted to the cancer site, caused apoptosis of cancer cells by phototherapy, and monitored therapeutic effects in situ. In addition, Mn-IR825 NMOPs was applied as a photothermal agent for light-sensitive targeted ablation of tumor tissue [71]. They have demonstrated that the nanoplatform has low cytotoxicity, good light stability, high photothermal conversion efficiency, and can be excreted quickly, avoiding long-term toxicity by in vitro and in vivo assay. Therefore, it has prospects for tumor imaging and treatment. Furthermore, Indocyanine Green (ICG) is a negatively charged polymethyl cyanine dye. Unlike cyanine dyes such as Cy3 and Cy5, it is under a higher absorption and emission wavelength. It allows deeper penetration than fluorescein angiography. However, due to its low solubility and low tumor specificity, a carrier material is urgently needed to load it to increase its clinical application. Cai et al. developed MOF@HA@ICG, which had a drug loading of 40% and was targeted to reach cancerous tissues through hyaluronic acid modification [50]. The system had minimal toxicity, good stability, and strong near-infrared absorption. More importantly, the nanoplatform enabled FL imaging, PAI (photoacoustic imaging), T2-weighted MRI, and PTT treatments. These supply the possibility for MOF-based PTT clinical applications.

**Figure 3:**
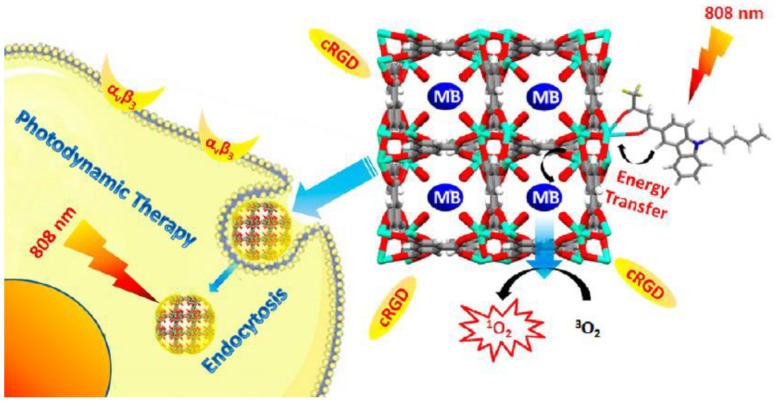
Schematic diagram of targeting of Eu(III) -based nanoscale metal organic framework to the tumor site and realized photodynamic therapy under the excitation of 808 nm near infrared light [20]. Reprinted (adapted) with permission from (Functionalized Eu(III)-Based Nanoscale Metal–Organic Framework To Achieve Near-IR-Triggered and -Targeted Two-Photon Absorption Photodynamic Therapy). Copyright (2018) American Chemical Society.

Photodynamic therapy (PDT) refers to the use of photodynamic effects for the diagnosis and treatment of cancer. It consists of three basic elements: oxygen, photosensitizer and visible light. The basic principle is that the photosensitizer enters the tumor tissue and is activated by light of appropriate wavelength to produce a photosensitivity reaction, which ultimately results in cell damage and death. For example, Zhu et al. reported low cytotoxic iron-porphyrin MOF modified by BSA and SA can trigger tumor photothermal therapy and photodynamic therapy even under hypoxic condition [42]. The authors found that a single source at 660nm induced greater damage to the tumor by PDT and PTT in these nanocomposites. Another example is that Nian and his colleagues have prepared a series of photosensitive nano drug delivery systems based on UiO-66 [11]. They achieved light-sensitive targeted therapy by modifying phthalocyanine (Pc) and Erlotinib (E). Experimental results show that the nanosystem has good anticancer activity. In addition, Jin and his team used isotope labeling on nanoparticles and found that ^64^Cu can achieve magnetic resonance imaging and photoacoustic tomography [72]. Finally, the cancer tissue is destroyed by the photothermal effect to achieve therapeutic effect. Tumor tissue temperature increased by 20 °C within 5 min. In Jia’s paper, HTHA (4,4,4-trifluoro-1-(9-hexylcarbazol-3-yl)-1,3-butanedione) could improve the penetration of infrared light into tissues and avoid tissue damage (Figure 3) [20]. MB is a photosensitizer that can produce singlet reactive oxygen under the illumination of near-infrared light. cRGD can target MOF to the specific location and improve the biocompatibility of drug-loaded system. All of these were allocated to one system and work together to increase the anti-tumor activity of MOF. It was necessary to demonstrate this platform had the best therapeutic effect at 808 nm. He and his coworkers synthesized a light-sensitive Zr(IV)-based Porphyrinic metal organic frameworks, through the modification of UCNPs, enhanced the production of singlet reactive oxygen species, thereby improving anti-tumor ability [47]. The cytotoxicity is the strongest under 980 nm near-infrared light, and the death rate of cancer cells reaches 80% within 20 min. However, there are still some shortcomings in photodynamic therapy that severely limit its clinical application, such as unprotected penetration ability, and only showing strong killing effect on superficial cancer tissues, which lead to PDT is mostly used for the treatment of skin cancer. In addition, photodynamic therapy is required to provide an aerobic environment, and tumor tissue is also in an anoxic environment due to its specificity, which also affects the effect of photodynamic therapy. Therefore, it is imperative to find strategies to overcome these problems, such as using near-infrared rays to penetrate deep tissues and loading substances in the drug-loading system that can improve the anoxic environment, such as catalase. Fang et al. designed and synthesized Lu@CoTCPP(Pd), an inner light integrated metal-organic framework to overcome the above difficulties [73]. This nanosystem achieved photodynamic therapy through the inner chemiluminescence resonance energy transfer (CRET) and had a curative effect on deep tumors.

Light-responsive targeting of cancer with MOF-based nano-therapeutics can diagnose and treat at the same time. Photoresponsive therapy can induce cancer tissue ablation, but its selectivity is low. When using this strategy, we are required to determine the location of the tumor, which reduces the efficiency of the treatment, and most of the drugs have been metabolically inactivated during prolonged circulation in the body. Therefore, most researchers nowadays are accustomed to using this strategy in combination with active targeting or other strategies to improve efficacy.

#### 3.3.3. Magnetic-Field-Responsive Metal Organic Frameworks-Based Targeted Anticancer Treatment

Magnetic-responsive treatment refers to the combination of drugs with magnetic materials (such as Fe_2_O_3_) that reach the tumor area under magnetic field guidance. Magnetic materials are not merely capable of guiding magnetic targeting but also as T2 contrast agents for magnetic resonance imaging. Thus, magnetic responsive therapy is a promising approach to cancer treatment. In this context, paramagnetic materials and superparamagnetic materials show great ascendancy.

For instance, Ke and coworkers first reported magnetic MOFs for targeted drug delivery [74]. It refers to the combination of Fe_3_O_4_ and MOFs to form magnetic MOFs. Fe_3_O_4_/Cu-based MOFs loading Nimesulide can be utilized to treat pancreatic cancer. It also can be utilized for magnetic resonance imaging and controlling drug release based on the nanoplatform. Although they failed to overcome their side effects, they provided a new idea for drug delivery for subsequent researchers. Immediately after, Yang et al. developed a magnetic-field-responsive drug delivery system based on Fe_3_O_4_/ZIF-8-Au25 (IZA) nanospheres for magnetic targeting, phototherapy and nuclear magnetic imaging [75]. The group verified through in vitro and in vivo experiments that the therapeutic effect under external magnetic field is significantly higher than that without magnetic field. In addition, CoFe_2_O_4_NPs@Mn-organic framework was published in Ahmad’s article [34]. Daunorubicin was successfully encapsulated in this nanocarrier. Subsequent experiments showed that the material can promote apoptosis of MCF-7 cells. In addition, it has controlled release ability and low cytotoxicity. The above experiments provide possibilities for the application of magnetic-field-responsive MOF. A composite material of graphdiyne and MOFs, Fe_3_O_4_@UIO-66-NH_2_/graphdiyne (FUGY), with superior ability for magnetic targeting, was designed and synthesized by Xue’s team [76]. This integrated nanosystem releases more drugs at the tumor site.

However, superparamagnetic iron oxides (SPIOs) are considered as a kind of negative contrast agent whose imaging effect is dark. There is another positive contrast agent based on Gd^3+^, but Gd^3+^ has greater cytotoxicity. Chelates are often used to stabilize Gd^3+^ and reduce its toxicity. Thus, Misty and colleagues prepared a polymer-modified Gd^3+^ MOF to eliminate the side effects of Gd^3+^ and used it for magnetic targeted imaging [45]. Growth inhibition studies were used to evaluate the safety of materials. The Gd MOF nanoparticles with the RAFT (reversible addition-fragmentation chain transfer) copolymer not containing MTX increased cell viability compared to Gd(III) chloride salt, 1,4-benzenedicarboxylic acid methylammonium salt and unmodified Gd MOF nanoparticles. The results show that the metal-organic framework modified by the polymer not only has excellent imaging effect, but also eliminates the toxicity of Gd^3+^. The combination of the nanocarrier with other functional substances is made possible by the introduction of the polymer. GRGDS-NH_2_ was successfully modified in this drug delivery system, which made it possible for this system to recognize α_v_β_3_-integrins and target to FITZ-HSA tumor cells. In addition, Kathryn and colleagues synthesize Mn NMOF for magnetic resonance imaging [77].

This strategy based on magnetic targeting can both diagnose and treat tumor. Of course, it also has the same problem as light-sensitive drugs, that is, the efficiency is relatively low, and researchers apply it more to the diagnosis of tumors.

#### 3.3.4. Targeted Drug Delivery Strategy Based on Thermosensitive MOFs

There are some metal organic frameworks that are sensitive to heat. We can use these temperature-sensitive metal organic frameworks to deliver drugs. Hyperthermia on the body can effectively stimulate the release of the drug by MOFs. This may be due to the fact that MOFs are prone to degradation at slightly elevated temperatures, or it may be due to a decrease in the force between MOFs and drugs under hyperthermia conditions. Xing and his group designed a temperature and pH dual responsive MOFs, Zn-cpon-1 [78]. The nanoparticles can be dual-targeted without any modification, which enables synthesize materials and load drugs simply and efficiently. The release rate of nanosystems increased with increasing temperature. Lin et al. synthesized ZJU-64 and ZJU-64-CH_3_, which enabled heat-responsive delivery of drugs [79]. These two materials released drugs more quickly and faster under the high temperature conditions caused by hyperthermia. This phenomenon was due to high temperature break host-guest interactions. Jiang and his team reported a nano-system with thermally responsive release drug [36]. ZJU-801, due to the introduction of the naphthalene moiety, showed a different release property from NU-801. ZJU-801 accelerates drug release rate with increasing temperature, while NU-801 shows burst release at different temperatures.

#### 3.3.5. Targeted Anti-Tumor Therapy Strategy Based on Ion-Responsive MOFs

Encapsulation of the drugs in the metal organic framework by ionic interactions tends to have a higher drug loading. This interionic force controls the release of the drugs. Yang et al. constructed a cationic metal organic frameworks (ZJU-101) to delivery diclofenac anions [80]. The drug delivery system controlled the release of the drug by exchange between the anions. Due to the interaction between ions, the drug loading of the nanosystem reached 0.546 g/g. The delivery system can release drugs faster under weakly acidic conditions. Wu and his team designed Fe_3_O_4_@UiO-66-NH_2_ [81]. They achieved ionic sensitive release drugs by modifying WP6 on the surface of the material. Under pathological conditions, local Ca^2+^ and Zn^2+^ levels in the body usually increased. These ions will stimulate the drug delivery system and promote drug release. Regardless, the advent of ion-responsive delivery systems provides new directions for targeted therapy.

#### 3.3.6. Redox Phase-Responsive-Metal Organic Frameworks-Based Targeted Anticancer Treatment

Redox-responsive release refers to the modification of a specific group on the surface of MOFs to encapsulate the drugs inside the materials. Only when certain conditions are met will the redox reaction be stimulated to expose the drugs and release the drug molecules. For example, Lei and his partner designed and synthesized a redox-responsive metal−organic framework, MOF-Zr(DTBA), to load curcumin for anticancer experiments [64]. Overexpressed glutathione (GSH) in tumor cells can cleave the disulfide bonds in this nano platform, and then lead to the release of curcumin.

### 3.4. MOF-Based Nanotherapeutics for Gene Delivery

As a biomarker, microRNAs usually express abnormally in cancer cells. Detection of overexpressed microRNAs can help diagnose cancer in time to avoid further deterioration or metastasis. The metal organic framework nanoparticles with gene transfer function can accurately detect over-expressed microRNAs in cancer cells. This enables us to provide appropriate treatment strategies early in the cancer which can improve the survival rate of cancer patients.

Metal-based organic matrix-based nanomaterials were examined as gene delivery vehicles by Yi et al. who first loaded the nucleic acid probe with ZIF-8 and delivered the nanoplatform to live cells [82]. Due to the pH sensitivity of ZIF-8, then this nanoplatform degraded in the acidic endosome to release the nucleic acid probe and Zn^+^. Zn^+^ acts as a cofactor for 8-17 DNAzyme and can be utilized to microRNA imaging to become a tool for tumor diagnosis. Additionally, Wu and colleagues exploited NMOF (UiO-66) nanoparticles (NPs) and fluorescence-labeled peptide nucleic acid (PNA) binding products to accurately and specifically detect the content of cells polypeptide miRNA and spatiotemporal changes in living cancer cells [44]. Peptide nucleic acid (PNA) has different fluorescence phenomena when combined with different substances, and it has no fluorescence when loaded on a metal organic skeleton nanocarrier. When the nanoplatform reaches the cancer cell region, it releases PNA and bind to the target miRNAs to generate fluorescence. Their team provides a very effective strategy for miRNA monitoring. Qiu bound five DNA probes to MOF1, which specifically recognize five microRNAs in gastric cancer cells [83]. After the DNA molecule is complementary to the target microRNA, the fluorescent molecule emits a signal, which provides us with immediate diagnostic information.

In addition, Chen et al. modified ATP aptamers or ATP-AS1411 hybrid aptamers on drug-loaded MOFs (Rhodamine 6G and doxorubicin loaded MOFs) [84]. ATP aptamer or ATP-AS1411 hybrid aptamer can be used as a switch to control drug release. When the drug-loading system recognizes ATP-expressing cancer cells, they can combine with ATP. Chen describes this behavior as ‘uncovering the hat’. This will advance the release of the drug. When the drug-loaded particles do not recognize the cancer cells, their caps will not open and the drug will be stably encapsulated in the system. The system is able to enter MDA-MB-231 breast cancer cells. It shows higher cytotoxicity than normal MCF-10A epithelial breast cells. Overexpression of some albumin receptors on tumor cells suggests that we can take advantage of albumin as a reactive transport reactor. Su et al. modified the AS1411 aptamer on the surface of the metal organic framework to target cancer cells [17].

The application of genes to the targeted therapy of MOFs enables precise localization and is much more efficient than active targeting. This strategy has become a hot topic of current research. However, there is also a need to defeat the problem of drug-loading systems being easily cleared by immunization.

### 3.5. MOFs-Based Bionic Immune Escape Strategy

In recent years, the rise of bionic technology has inspired researchers in the field of biomedicine. The application of biomimetic technology based on MOFs in anticancer treatment can effectively solve a series of problems stemming from other strategies. Of course, this strategy has stricter requirements on the particle size of MOFs. Biomimetic techniques which mean modifying tumor cell membrane fragments on the surface of MOFs have been mentioned previously. A biomimetic nanoplatform (CAT-PS-ZIF@Mem) modified by cancer cell membrane fragments was developed by Cheng to enable self-providing O_2_ [85]. Cancer cell membrane fragments helped rapid localization and long-term cycling of nano drug-loading systems, improving bioavailability. The authors hoped to be able to increase the oxygen content of tumor tissues by catalase. Confocal Laser Scanning Microscopy and UV–Vis experiments also verified that under the action of catalase, endogenous hydrogen peroxide decomposed to produce O_2_. The study also found that Al(III) phthalocyanine chloride tetrasulfonic acid (AlPcS4) would self-quench, and its ability to produce ^1^O_2_ was greatly enhanced by being encapsulated with MOFs. This strategy of bringing functions together on one platform greatly enhanced the efficiency of cancer prevention. Li and coworkers exploited a relatively novel approach for multi-targeted anti-cancer research [61]. It acted on cancer tissues that combined a biomimetic theranostic O_2_-meter with photodynamic therapy. Since porphyrin needed to consume oxygen, the oxygen content of the tumor area would decrease as the treatment progresses, which would lead to a decrease in treatment efficiency. The drug-loaded nanosystem could detect the content of O_2_ in the tumor area on the one hand, and achieve immune escape on the other hand, and which jointly improve the anti-cancer efficiency. A similar report was issued by the same team who used biomimetic technology to achieve high-efficiency, precise targeting and avoid removal by the immune system [29]. They increased the oxygen content of the tumor microenvironment by loading reactive oxygen species and catalase on the MOFs, improving the efficiency of photodynamic therapy while achieving starvation therapy. A series of experiments in vitro and in vivo confirmed the system’s excellent anti-cancer ability. Furthermore, they reported another anti-cancer platform-TPZ@PCN@Mem based on the bionic principle [86]. It described that when porphyrin was used for phototherapy. Oxygen was consumed to cause a hypoxic environment, which deteriorated the therapeutic effect. However, the team did not improve the hypoxic environment but made the drug system utilized this hypoxic environment to fight cancer. There were reducing drugs that kill tumor cells in a hypoxic environment. Therefore, they proposed modifying MOFs with tirapazamine (TPZ). Depending on their report, TPZ@PCN@Mem could damage cancer tissue with minimal side effects. Bionic technology has unparalleled superiority in the field of anti-cancer. Accurate drug delivery, immune escape, long-acting cycle, and other advantages made bionics technology arouse the interest of many researchers, and became a hot topic in recent researches. Researchers often combined biomimetic techniques with other targeted strategies to improve the targeting capabilities of nanosystems and to improve treatment outcomes. Wan simultaneously demonstrated biomimetic technology and photodynamic therapy on a nanoplatform. Considering the limitations of photodynamic therapy (resistance to reactive oxygen species and unstable treatment efficiency), gas therapy was added to the platform [87]. On the one hand, L-Arg activated photodynamic therapy under near-infrared conditions, and on the other hand, L-Arg acted as an oxidant to cause nitrification to produce NO. Therefore, treatment could be continued under normal conditions or under hypoxic conditions. Coupled with the help of cancer cell membrane fragments, drug-loaded system could achieve targeted therapy and avoid excessive immune clearance. Experimental results showed that 4T1 tumors were completely ablated with negligible side effects. This suggests that we can rely on multi-targeted therapy to overcome the drawbacks of a single treatment, which can improve the treatment effect.

### 3.6. MOFs-Based Core-Shell Nanomedicine Carriers

It is worth to mention that appropriate modifications to MOFs using other materials can effectively take advantage of various materials, and its shortcomings can be ignored. Chen et al. synthesized ^89^Zr-UiO-66/Py−PGA-PEG-F3 for positron emission tomography of tumors [41]. This technique was utilized because it was more penetrating and more sensitive than optical imaging. Py-PGA-PEG was capable of providing a group for binding to a peptide ligand (F_3_) and a nucleoside. Nucleosides can target tumor cells to improve the therapeutic effect of drugs on cancer and reduce biological toxicity. The nanoplatform has also been shown to be pH-sensitive, with a low release rate under normal physiological conditions, a slightly better release rate in the tumor environment, and the best release effect under tumor cytoplasmic conditions, with a cumulative release rate of approximately 37.06% in 2 weeks. In spite of this, its release conditions had yet to be improved to improve release rate and utilization. In vitro and in vivo experiments showed that the material had excellent effects for radiotherapy of cancer, and no toxicity was noted in the body. This study can encourage other researchers to study further radioactive MOFs. Furthermore, a core-shell nanoparticle UCNPs@MOF NCs was developed by Deng et al. who modified AS1411 aptamer on the surface of the particles to specifically recognize the nucleolin on the surface of tumor cells [48]. This nanoplatform could rely on UCNPs for optical imaging and convert near-infrared light into visible light to minimize light damage and achieve greater penetration. In vitro cell experiments found that the substance acts on MCF-7 cells (human breast cancer cells) to produce green fluorescence, while 293 cells (human embryonic kidney cells) have no positive results. The study also found that UCNPs@MOF NCs had a greater affinity for cancer cells than simple target receptors, which meant that there were other beneficial effects for materials and cancer cells. Meanwhile, the composite material could also achieve pH sensitive release of the drug, that was to say, the slightly acidic environment of the tumor tissue could cause degradation of the system to release doxorubicin. Considering the combination of UCNPs with mesoporous silica or hydrophilic polymers leaded to a decline in photo-imaging function, and it was become a necessity to find new carrier materials. The metal organic frameworks can load UCNPs without affecting the optical function of UCNPs, and at the same time achieve drug loading. Therefore, there were several reports on the combination of UCNPs and metal organic frameworks for cancer treatment. What is more, multi-functional combination showed a stronger anti-cancer effect. Another similar report was published by Angshuman, who used the same principles to synthesize the core-shell nanomedicine carriers (UCNPs@ZIF-8/FA/5-FU) mentioned above using different materials [49]. Similarly, the drug delivery system enabled pH-sensitive release and fluorescence image, and the system could easily reach cancerous tissue due to folic acid modification. UCNP@UIO-66(NH_2_)/FA/DOX was also synthesized using the same principle [88]. Angshuman used this nanoparticle for breast cancer treatment. The in vitro cell experiments showed that the particles were effective against the MDA-MB-468 cells. The similar material had a high drug loading capacity (DOX), which satisfied the pH-responsive drug release, actively targeted cancer cells through folic acid, and achieved up-conversion luminescence. Its in vivo experiments have yet to be further studied. The development of cancer therapy has been progressing, and people’s pursuits are also growing. Using a variety of materials to fight cancer is undoubtedly a cost-effective way. Of course, we just have to consider the reactions that these strategies produce when we use them in the body. Therefore, deeper research needs to be done in the future.

### 3.7. Multi-Targeted Response of MOF Nanomaterial for Anticancer Treatment

Each of these methods has its limitations, and more researchers are focusing on multi-targeted anti-cancer. They combined several targeted strategies on a nano-platform to achieve precise treatment and eliminate the shortcomings of each strategy.

**Figure 4:**
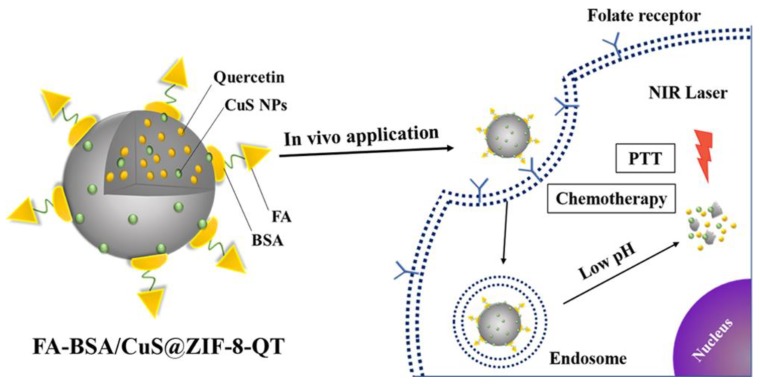
Schematic illustration of the combinational therapy platform based on folic acid (FA)−bovine serum albumin (BSA)-modified ZIF-8 (FA−BSA/CuS@ZIF-8-QT) [90]. Reprinted (adapted) with permission from (CuS@MOF-Based Well-Designed Quercetin Delivery System for Chemo–Photothermal Therapy). Copyright (2018) American Chemical Society.

The most common is to combine active targeting with other targeted strategies. Armed with this strategy, Song et al. applied both pH response and light response to ZIF-8 and synthesized ZnPc@ZIF-8 and ZnPc@ZIF-8/CTAB [14]. The targeting system released photosensitizers around tumor cells and increased photodynamic anticancer efficiency by producing excessive ROS. Such a strategy was mentioned in Chen’s report that the modification of folic acid to pH-sensitive ZIF-8 enabled dual targeting and efficient delivery of Epigallocatechin-3-gallatea to target cells. Moreover, M-NMOFs was a superparamagnetic nanoparticle synthesized by Shalini through the AOT (Aerosol OT) microemulsion method [28]. Meanwhile, this M-NMOFs was utilized to wrap the doxorubicin and the photosensitizer methylene blue. The nanoplatform was transferred to the target area under the intervention of an external magnetic field to release doxorubicin. Photosensitizers also exhibited photodynamic effects under light, which greatly enhanced cytotoxicity. Thus, this nano transport system combined magnetic targeting and photoresponse to enhance anticancer effects. This system provided some reference for additional researchers. For the first time, Angshuman used folic acid to modify magnetic nanocarriers encapsulating paclitaxel and the fluorescent molecule rhodamine B isothiocyanate (RITC) [10]. Therefore, paclitaxel could be well absorbed by liver cancer cells, and fluorescence analysis and magnetic resonance imaging would be performed through this nano platform. Folic acid-modified MOFs have improved the performance of the original MOFs which can’t achieve precisely targeted positing and efficient therapy. Similarly, folic acid-modified multifunctional nano drug-loading system also appeared in Liu’s research [46]. As a photosensitizer, chlorine e6 (Ce6)-labeled CaB substrate (Ce6-peptide) provided photodynamic therapy. Cam was employed as an anticancer drug for chemotherapy. Folic acid as a ligand could specifically recognize cancer cells carrying folate receptors and achieve localization. Combined with various treatment methods, CPC@MOF improved the efficiency of cancer prevention. Kin et al. also used folic acid modification for active targeted therapy [89]. A calcium zoledronate (CaZol) and a polyethylene Glycol (PEG) formed a nano-metallic framework of core-shell structure, and CaZol was not only a structure of a metal organic framework but also an anticancer drug. Folic acid-modified nMOFs were more potent than Zol alone in vitro and in vivo. The nano-platform was also pH sensitive. That was to say it was stable at pH 7.4, and would be internalized by the cells in the tumor area to release Zol. Shi et al. also prepared a pH responsive and folic acid induced dual targeting formulation FA-PEG/CQ@ZIF-8 [13]. The same strategy was also included in Dong’s report [33]. The folic acid was modified on a pH-sensitive metal organic framework to obtain FA/5-FU@MOF-808 and FA/5-FU@NH_2_-UiO-66. The nanomaterial also had good targeting properties. In addition, as an active targeting agent, folic acid−Bovine serum albumin (FA−BSA) was modified on ZIF-8, which was also loaded with CuS and quercetin (Figure 4) [90]. The nano-platform can actively transport the photothermal agent and the chemotherapeutic drug to the cancer tissue. Fluorescence imaging revealed that the drug was successfully internalized by cancer cells, and the cancer tissue was ablated by near-infrared irradiation. The combination of chemotherapy and PTT, coupled with the targeting function of FA-BSA, made the anti-cancer effect of the drug system far greater than the single anti-cancer treatment. Wu also did a similar job, which was to use PTT in combination with chemotherapy to complement each other and improve the anti-cancer ability of the drug-loading system [91]. At the same time, folic acid acted as a guide to transport the drug-loaded particles to the target site. The difference was that the system had pH and temperature sensitive properties due to the addition of pillararene-based pseudorotaxanes. Of course, in addition to folic acid, other molecules can be utilized to induce active targeting. Dong et al. used RGD to recognize the α_v_β_3_ receptor on the surface of cancer cells and synthesized RGD@CPT@ZIF-8 [54]. At the same time, the nanoplatform was also sensitive to pH. It did not release in a neutral environment and released 75% of the drug within 24 h in a weak acid environment. In addition, DOX@MOFs-Glu was synthesized by Zhang and his colleagues who combined pH-responsive drugs, active targeting and CT imaging [55]. The glucose specifically recognized the glucose-transported protein (GLUT1) overexpressed on the surface of tumor cells. Gd^3+^ ions can contribute to achieving magnetic resonance (MR) imaging. 5-boronobenzene-1, 3-dicarboxylic acid (BBDC) could bind to glucose and be polymerized or decomposed in different pH environments to control drug release. The nano-platform had excellent biocompatibility due to the addition of glucose, and was not readily metabolized in the body. In summary, it will produce a stronger and more effective lethality on tumor tissues to combine active targeted therapy with other types of anticancer methods. It has become a more conventional multi-targeted anti-cancer approach in recent years. Of course, there are numerous other multi-targeted strategies for efficient and accurate cancer treatment. ZIF-67/Fe_3_O_4_/DOX was obtained by a simple method, which could be targeted to the tumor tissue under the action of an external magnetic field. Then the drug-loading system released the drugs under the action of H_2_O_2_ catalysis and water effect [92]. This versatile MOFs nano-platform could not only perform excellent positioning functions, but also improve the therapeutic effect together with chemotherapy.

If the diagnosis and treatment of the tumor can be completed in one step, it will provide greater convenience. In Gao’s paper, magnetic mesoporous nanomaterial Fe-MIL-53-NH_2_, chemotherapeutic drug 5-fluorouracil (5-FU) and the fluorescence imaging agent 5-carboxyfluorescein (5-FAM) were pooled in a nanoplatform with folic acid for active targeted modification [31]. Although using common modifications, this identical group synthesized a nano platform that integrated pH sensitivity, fluorescence imaging, and folate receptor-specific recognition to actively targeting [32]. ZIF-8 was a pH-responsive material, folic acid was used to actively target cancer cells, 5-FAM was used as a fluorescent agent to monitor drugs, and chitosan could improve the affinity of folic acid and 5-FAM with ZIF-8. The difference was that Gao et al. experimentally verified the localization of folic acid. It was found that the folate-negative nanosystem had insufficient affinity with tumor cells, and the nano-platform modified by folic acid can bind well to tumor cells, but can not enter normal cells. In addition, Du et al. synthesized ZIF-8, then utilized Fe^2+^ to absorb this MOFs [93]. Interestingly, in the tumor environment, low pH and high GSH content, Fe^2+^ was oxidized to superparamagnetic Fe^3+^, ZIF-8 was degraded into fluorescent agent ZnO. What is more, there was no such change in the normal cellular environment. Thus, this strategy can be exploited for early cancer diagnosis. In addition, new indocyanine green (IR820) and Cytarabine (Ara) were co-loaded in ZIF-8 by Zhang et al. to achieve chemotherapy, fluorescence imaging and PTT [18]. This strategy also combined diagnosis with treatment. Active targeting has become one of the functions of this platform due to the surface modification of hyaluronic acid. In addition, Zhang and colleagues synthesized AuNS@MOF-ZD2 nanocomposites, which combined PTT and MRI [94]. The nanomaterial could produce thermal effects under the irradiation of 808nm light which can induce apoptosis of cancer cells. Magnetic resonance imaging would diagnose and observe the therapeutic effects. The hemin and Ni were assembled together to form an enzymatically active metal organic framework, which could undergo a redox reaction [51]. By folic acid modification, the MOFs would detect the presence of cancer cells very sensitively, and the generated reactive oxygen species also had a strong killing effect on MCF-7 cells. This strategy could monitor the prognosis of the tumor in real time, so that the treatment plan could be adjusted at any time.

Although active targeting can deliver drugs to tumor tissue, it is more or less metabolized or pre-released before reaching the tumor tissue. As a result, the researchers demonstrated a nanosystem that released drugs only in specific areas. There was a door in this drug-loaded nanosystem. The door was used to lock the drugs in the MOFs. When it reached the tumor area, the door opened and the drug was released. Two different stimulating drug-released MOFs were presented in Chen’s report [95]. Both were nucleic acids-based multifunctional MOFs. Nucleic acid double-strand can cover the drug-loaded MOFs to form a lock and control the release of the drug. The two nanoparticles surface-modified with the AS1411 aptamer bond to ATP of tumor cells and promoted the internalization of drug-loaded particles by cancer cells. One of them was sensitive to pH. When the drug-loading platform was in a weakly acidic tumor microenvironment, the double strands opened, prompting drug release to take action on cancer cells. The other was a metal ion-dependent drug release system. Only in the presence of metal ions of DNAzyme and substance complexes, the double strand was opened and released the drug. This unique drug delivery system accurately controlled the drug’s internalization by cancer cells, producing strong cytotoxicity. Based on the same strategy, Zhao modified the metal organic framework with nucleolin-specific AS1411 aptamers and encapsulated porphyrins and doxorubicin, which simultaneously achieved pH-sensitive, active targeting and photodynamic therapy [96]. Currently, Wu has also developed a controlled release strategy based on MOFs, which used water-soluble carboxylatopillar [6] arene (WP6) as a valve to control drug release [81]. When the drugs were encapsulated in a nanosystem, under normal conditions, the valve of the system was closed, the drugs cannot be released, and the valve would open when the drug-loaded particles reached the designated site, and the drug would be released. The conditions under which the valve was opened were in a slightly acidic environment or at a slightly higher temperature or a high iron content. In view of the multi-functional nano-targeting platform has become a hot topic of current researches. The Fe_3_O_4_@UiO-66@WP6 synthesized by Wu was also utilized Fe_3_O_4_ as core, which constituted a magnetically sensitive drug release system. The drug release ability, safety, and effectiveness in vitro of the system were evaluated, and it was regarded as having application prospects. However, the study lacked in vivo studies. Whether the system can be used before being metabolized has to be proven. This strategy can effectively reduce the release of drugs outside the target area to reduce adverse reactions and improve the therapeutic effect.

## 4. Possible Challenges of MOFs Application in Cancer Therapy

### 4.1. Quality Control: from Small-Scale Production in Laboratories to Large-Scale Industrial Production

As mentioned above, MOFs are excellent drug carriers. However, at present, the researches on the biomedical performance of MOFs remain in small-scale production and experiment in the laboratory. When MOFs are synthesized in mass production, their quality is often difficult to control which may lead to the changes in material size, pore size, etc. Drug loading capacity and release rate will also be affected. Therefore, the development of stable and controllable MOFs is one of the most serious challenges.

### 4.2. Toxicity and Biocompatibility

Despite the incomparable advantages, more attention needs to be paid to the in vivo studies of MOFs, including biocompatibility and toxicity. As degradable materials, the mechanism and metabolic processes of MOFs in the body need more data to understand. In recent years, a large number of studies on the in vitro cytotoxicity of MOFs have been reported [27,35,45,55,56,58,59,60,61,77]. Although these data show that MOFs have good biosafety at a given dose, cell models do not demonstrate that they still display the same biocompatibility in the body. Although some studies have reported anti-tumor effects of MOFs-based drug delivery systems in experimental animals, studies on their metabolism and toxicity have rarely been explored.

Zhang et al. used breast cancer nude mice as a model to study the metabolism of Fe3O4@C@PMOF in nude mice [97]. The nanoparticles can be used for fluorescent imaging in vivo. The researchers observed fluorescent spots in the liver and lymph nodes, demonstrating that the nanoparticles can participate in both blood circulation and lymphatic circulation. Subsequently, the tumor area became the tissue with the brightest fluorescence intensity. Within 8 days, the nanoparticles were eliminated from the body through feces. The injected mice behaved normally and the weight did not decrease remarkably. Eight days after injection, no pathological changes were noticed for the main organs of mice. It shows that the nanoparticles have good biocompatibility. Tarek et al. analyzed the in vivo toxicity of iron(III) MOFs [98]. All studied parameters (serum, enzyme, histology, etc.) were consistent with low acute toxicity. The nanomembrane is isolated by the liver and spleen, and then further biodegraded into iron and organic carboxylic acids, and is directly cleared in urine or feces, maintaining intact the iron homeostasis. This shows iron(III) carboxylate MOFs nanoparticles are biodegradable and non-toxic. Chen and his team studied the in vivo biosafety of a porphyrinic MOF nanoplatform [99]. They evaluated the toxicity of the nanoplatform to the main organs, tissues, and blood of mice. All indicators are normal, which indicates that the nano system has good security. Ma et al. also demonstrated the biosafety of quercetin-modified Zr-MOFs through similar experiments [100]. The nanoparticles did not show organ and blood toxicity. Wang and his colleagues studied the long-term toxicity of porphyrinic MOF Nanodots in the body [101]. They found that these nanoparticles had extremely low systemic toxicity and could eventually be cleared through the kidneys.

Anyway, the clinical progression of MOFs still requires a large number of experiments to study its biological processes and biosafety in vivo. One need to focus not only on the novel and many design strategies to treat cancers, but also on clearance and toxicity.

### 4.3. Avoid Drug Release or Immune Clearance Before Reaching the Target Site

The chemotherapeutic drugs usually cause serious adverse reactions to the body. Therefore, it is important to improve the stability of the targeted drug delivery system so that they were not released outside the tumor and cause severe adverse reactions to the body. We also are required to prevent the drug-loaded particles from being cleared by the immune system before reaching the target site. Taken into account this, biomimetic techniques coated with cell membranes have emerged, but this technique has higher requirements for materials. Thus, in order to obtain a MOFs-based drug delivery system suitable for clinical applications, we still require considerable development from the synthesis to quality control, as well as in vivo process monitoring. Anyway, MOFs-based drug delivery system has shown an unprecedented advantage.

## 5. Conclusions and Perspectives

We summarized the research reports on MOFs in tumor targeted therapy in recent years, and reviewed the tumor targeted therapy of MOFs from four aspects: passive targeting, active targeting, physicochemical targeting (pH response, light response, magnetic response, gene targeting), and multi-targeting, in order to provide reference and help for the research of MOFs in the precise treatment of tumors and other difficult diseases.

The adjustable pore size, biodegradability, and biocompatibility of MOFs make them ideal as drug delivery systems. However, ordinary MOFs-based drug-loaded granules usually fail to achieve long-term circulation in the body, which is metabolized prematurely by the body or cleared by the immune system. These are only to reduce the efficacy. More seriously, when the drug-loaded particles are in the process of circulation, the premature release of the chemotherapeutic drugs will make these drugs toxic to normal tissues. Excitingly, the structure of MOFs allows surface modification for targeted delivery. Targeting strategies can maximize efficacy and minimize adverse effects. Usually a strategy is not sufficient for the purpose of delivering drugs efficiently. The emergence of multifunctional MOFs can make up for the shortcomings of a single targeting. Multi-targeted response MOFs nanoparticles have become the most commonly used strategy. We can also refer other materials to MOFs to absorb a wide range of advantages. In any case, we always believe that MOFs will be able to demonstrate their skills in the biomedical field.

Although there are still many challenges for MOFs to be used in actual production, such as unstable quality and unclear drug metabolism. We firmly believe that through our unremitting efforts, these problems will be solved in the near future. There is no doubt that targeted delivery systems based on MOFs are one of the most promising biomedical applications. We look forward to the day when we can defeat tumors, which will benefit all humanity.

## Figures and Tables

**Table 1:** Synthesis and functionalization of metal organic frameworks.

Drug Delivery System	Synthetic Method	Loading Capacity	Release Rate	Achievement	Ref.
DOX @ZIF-8	One-pot synthesis	20%	95% (pH 5–6, 37 °C, 7–9 days)	pH-responsive	[26]
PEG-FA/(DOX+VER)@ZIF-8	One-pot synthesis	8.9% (DOX) 32% (VER)	27.37% (DOX) 76.48% (VER) (pH 5, 37 °C, 24 h)	pH-responsive, Overcoming multidrug resistance	[27]
5-FU+DOX @ZIF-90	Ultrasonic stirring	36.35% (5-FU) 13.5% (DOX)	95% (5-FU, 15 h) 91% (DOX, 25 h) (pH 5, 37 °C)	pH-responsive, Combination therapy	[19]
DOX @ZIF-8 NTs	Template-directed synthesis	350% (drug/mo-fs)	72% (DOX) (pH 5, 37 °C, 50 h)	pH-responsive, Ultra-high drug loading and long-acting cycle	[22]
FA/5-FU @IRMOF-3	Solvothermal method	20.4%	68% (37 °C, 96 h)	pH-responsive, Active targeting	[9]
DOX @TTMOF	One-pot synthesis	14.3%	78% (10 mM DTT, pH 7.4, 37 °C, 140 h)	pH-responsive, Redox responsive	[15]
PD/M-NMOF	AOT microemulsion method	4.3% (MB) 0.69% (dox)	72% (MB) 95% (Dox)	Magnetic-responsive Light-responsive	[28]
PTX/Fe_3_O_4_@IRMOF-3	mixed solvent solvothermal method	12.32%	65% (pH 7.4, 37 °C, 100 h)	Magnetic-responsive	[10]
mCGP	solvothermal method	13.5% (glucose oxidase and catalase)	/	Starvation and Photodynamic Therapy	[29]
MB @THA-NMOF-76 @cRGD	rapid microwave-assisted method	3 ug/mg (MB)	/	Light-responsive, Active targeting	[20]
Gd-MTX NCP	microwave heating	79.1% (MTX)	100% (pH 7.4, 37 °C 192 h)	Active targeting	[30]
Fe-MIL-53- NH_2_-FA-5- FAM/5-FU	a reflux method at low temperature	23%	the gentle release for 25 h in pH 7.4 for 20 h in pH 5	Light-responsive, Magnetic-responsive Active targeting	[31]
ZIF-8/5-FU @FA-CHI- 5-FAM	solvothermal method	51%	complete release (pH 7.4, 37 °C, 45 h pH 5, 37 °C, 21 h)	Light-responsive, pH-responsive, Active targeting	[32]
FA/5-FU @MOF-808	stirring-reflux method	38.42%	60–70% (pH 5, 37 °C, 24 h)	pH-responsive, Active targeting	[33]
FA/5-FU@ NH_2_-UiO-66	stirring-reflux method	30.26%	60–70% (pH 5, 37 °C, 24 h)	pH-responsive, Active targeting	[33]
CoFe_2_O_4_@Mn-MOF	layer to layer method	75 ± 1.22% (Encapsu-lation efficienc-y)	55% (pH 7.4, 37 °C, 20 h)	Magnetic-responsive	[34]
BSA/Cu/NQ NP	protein-nanoreactorv method	13.6%	/	Active targeting	[35]
Fe-soc-MOF@PPy	The liquid-solid- solution (LSS) method	15%	/	Light-responsive	[21]

**Table 2:** The molecular formula of the metal organic frameworks (MOFs) that appear in this article.

Classifications	Abbreviations	Examples	The Molecular Formula	Ref
Isoreticular Metal Organic Frameworks	IRMOF-n	IRMOF-3	C_24_H_5_N_3_O_13_Zn_4_	[9]
Materials of Institute Lavoisier	MIL-n	NH_2_-MIL-53 (Fe)	C_8_H_6_NO_5_Fe	[31]
Zeolitic Imidazolate Frameworks	ZIF-n	ZIF-8	C_8_H_10_N_4_Zn	[27]
		ZIF-90	C_4_H_4_N_2_OZn	[12]
University of Oslo	UiO-n	NH_2_-UiO-66	C_48_H_30_NO_32_Zr_6_	[11]
Zhejiang University	ZJU-n	ZJU-801	C_12_H_4_O_32_Zr_6_	[36]

**Table 3:** Targeting strategies for metal organic frameworks.

Targeting Cancer Cell				
Drug	Target	Target Cell Line	Targeting Type	Ref.
BSA/SAs@MOF	CA IX	4T1 cancer cells	Light-responsive	[42]
Caspase-FA/TMPyP@MOF	FRs	HeLa cells	Light-responsive	[43]
FA/5-FU @IRMOF-3	FRs	HeLa cells, lung adenocarcinoma A549 cells, KB cells	Active targeting	[9]
PNA@UiO-66	miRNAs	MDA-MB-231, MCF-7	Gene-responsive	[44]
Polymer-Modified Gd MOF	α_v_β3-integrins	FITZ-HSA tumor cells	Magnetic-responsive Light-responsive	[45]
CPC@MOF	CaB	HeLa cells	Light-responsive	[46]
mCGP	(4T1) cancer cell membrane	4T1 cancer cell, B16F10 cells, HepG2 cells, COS7 cells	Starvation and Photodynamic Therapy	[29]
MB @THA-NMOF-76 @cRGD	α_v_β_3_-integrins	HeLa cells, A549 cells	Light-responsive, Active targeting	[20]
Gd-MTX NCP	sigma receptors	Jurkat ALL cells	Active targeting	[30]
Zr(IV)-based porphyrinic MOF–UCNP	epidermal growth factor receptor	The MDA-MB-468 cells	Gene-responsive, Light-responsive	[47]
Fe-MIL-53- NH_2_-FA-5- FAM/5-FU	FRs	MGC-803 and HASMC cells	Light-responsive, Magnetic-responsive Active targeting	[31]
ZIF-8/5-FU @FA-CHI- 5-FAM	FRs	MGC-803 cells	Light-responsive, pH-responsive, Active targeting	[32]
UCNPs@MOF- DOX-AS1411	nucleolin	MCF-7 cells	Light-responsive, pH-responsive, Active targeting	[48]
UCNPs@ZIF-8/FA/5-FU	FRs	HeLa cells, mouse fibroblast (L929) cells	Light-responsive, pH-responsive, Active targeting	[49]
MOF@HA@ICG	CD44	MCF-7 cancer cells	Light-responsive, Active targeting	[50]
Fe-soc-MOF@PPy	/	4T1 cancer cells	Light-responsive	[21]
FA@Ni-hemin metal organic framework	FRs	MCF-7 cancer cells	Active targeting, Redox responsive	[51]
PEG-FA/PEGCG@ZIF-8 NPs	FRs	HeLa cells	pH-responsive, Active targeting	[52]
Streptavidin/GOx@ZIF-8-AuNCs	biotinylated antibody against galectin-4	colorectal cancer, breast hepatocellular carcinoma, gastric cancer, etc.	Active targeting, Light-responsive	[53]
RGD@CPT@ZIF-8	α_v_β_3_ receptor	HeLa cells	Active targeting, pH-responsive	[54]
DOX@MOFs-Glu	glucose-transported protein (GLUT1)	HeLa cells	Active targeting, pH-responsive, the magnetic resonance (MR) imaging	[55]
